# Reconfigurable Split Ring Resonators by MEMS-Driven Geometrical Tuning

**DOI:** 10.3390/s23031382

**Published:** 2023-01-26

**Authors:** Angelo Leo, Alessandro Paolo Bramanti, Domenico Giusti, Fabio Quaglia, Giuseppe Maruccio

**Affiliations:** 1 Omnics Research Group, Department of Mathematics and Physics “Ennio De Giorgi”, Institute of Nanotechnology CNR-Nanotec, INFN Sezione di Lecce, University of Salento, Via per Monteroni, 73100 Lecce, Italy; 2 System Research and Applications, Silicon Biotech, Lecce Lab, STMicroelectronics S.r.l., c/o Ecotekne, Via per Monteroni 165, 73100 Lecce, Italy; 3 Analog MEMS and Sensors Product Group, STMicroelectronics S.r.l., Via Tolomeo 1, 20100 Cornaredo, Italy

**Keywords:** split ring resonator, MEMS, metamaterial, dynamic tuning, piezo actuator

## Abstract

A novel approach for dynamic microwave modulation is proposed in the form of reconfigurable resonant circuits. This result is obtained through the monolithic integration of double split ring resonators (DSRRs) with microelectromechanical actuators (MEMS) for geometrical tuning. Two configurations were analyzed to achieve a controlled deformation of the DSRRs’ metamaterial geometry by mutual rotation or extrusion along the azimuthal direction of the two constituent rings. Then, the transfer function was numerically simulated for a reconfigurable MEMS–DSRR hybrid architecture where the DSRR is embedded onto a realistic piezo actuator chip. In this case, a 370 MHz resonance frequency shift was obtained under of a 170 µm extrusion driven by a DC voltage. These characteristics in combination with a high Q factor and dimensions compatible with standard CMOS manufacturing techniques provide a step forward for the production of devices with applications in multiband telecommunications and wireless power transfer and in the IoT field.

## 1. Introduction

Reconfigurable devices, circuits and metamaterials are a major trend in current research in physics and engineering [1] with applications ranging from ICT [2] to sensors and monitoring technologies [3]. In this respect, metamaterial-inspired architectures [4] and split ring resonators (SRRs) attracted large interest [5,6,7]. On one side, their response can change as a function of an external condition, and this is relevant for the implementation of sensors exhibiting high performances and ultimate sensitivity [8,9,10]. On the other, their output can be tuned acting on a control parameter and such versatile modulation can enable relevant ICT applications [11,12]. For example, they can be used for implementing devices and filters for telecommunications and GHz applications in addition to technologies based on surface acoustic waves with piezoelectric materials or spin waves/ferromagnetic resonance within ferro/ferri-magnetic materials [13,14,15,16]. Present research efforts in this field focus on the increase in the transmission band, as well as the raise in the sensitivity and performance of signal transmission, in addition to further broadening the range of applications.

Given their small size, versatility, reusability and high sensitivity, split ring resonators (SRRs) are widely used in different types of sensors/devices with operating frequencies ranging from optical to microwave (MW) regime. For example, SRR-based platforms for THz science and applications were reported thanks to the optimization of reliable fabrication methods [17], which led to a significant increase in performance [18]. In this case, and in optical regime, a challenge currently being faced is the tunability of these devices, for which various strategies have been adopted, including those based on the use of electric actuators [4,19] and flexible substrates [20].

The same goals are of significant interest for metamaterial resonators working in the MW range, which are already widely used in their non-tunable form. Indeed, in industrial and environmental monitoring and biomedical fields, SRRs have been used for the evaluation of the concentration of ethanol and hydrocarbons dissolved in water [21,22], of oil matrices [23], pH [24] and microparticle flows [9,25,26]. Furthermore, they are suitable for integration within microfluidic platforms [27,28]. On the other hand, their high sensitivity to changes in dielectric properties makes them suitable for impedance-based sensing and biological tissues analysis [29,30,31]. For the same reasons, in combination with dielectric impedance spectroscopy [32,33,34,35,36], they provide convenient methods for the dielectric characterization of materials, exploiting resonant cavity perturbation methods [37,38,39]. SRRs can also enable near-field MW imaging [40,41], which is important for non-destructive 2D surface mapping of relative dielectric permittivity and the characterization of composite materials.

Structural monitoring is another sector of relevant impact due to the sensitivity of SRRs to small displacements [8,42,43]. In the telecommunications field, the use of SRRs regards wireless power transfer (WPT) [44] and modulation of the signal transmission band [45] (i.e., for WLANs [46] and wearable devices [47]), through either the definition of filters or the development of broadband and multiband devices [48,49]. Clearly, a dynamic tuning of the SRR response and its resonance frequency would be highly desirable for enabling novel opportunities for telecommunications, such as enhancement of isolation in higher frequency bands and access to multiband satellite and radar communications.

In this work, we propose novel reconfigurable split ring resonators by MEMS-driven geometrical tuning achieved through the monolithic integration of the two components, with applications for modulation of MW electromagnetic radiation from *S* band to *K* band. In the literature, the static response of SRRs has been engineered by fabricating them with different geometries—e.g., varying the relative position of the two rings by mutual rotation or linear displacement in double concentric structures [50,51]. This tuning is static, however, i.e. decided once for all in the design phase. The ability to dynamically tune on request the response of a single, reconfigurable device would be a significant step forward in terms of technology since this progress would enable further applications where a user-defined modulation is needed to adapt the characteristics to changing requirements. So far, a few solutions are available for electronic tuning by means of solid-state varactor or switching via PIN diodes [52,53] typically placed on SRR slits. In these cases, signal rectification and then frequency modulation occur through the injection of an electromagnetic (resonant) signal and a solid-state device actuation signal. The implementation of electronic tuners can also be carried out with MEMS, and some solutions have been presented, essentially based on the use of MEMS-based switches [54], varactors [55,56] and capacitors [57].

The novelty of the present work consists of the proposal of geometrically tunable split ring resonators capable of dynamic modulation in the MW range by integrating double SRRs (DSRRs) with currently available MEMS technologies for actuating both the mutual linear displacement and rotation of the two rings. We report a systematic study of the dependence of the transfer function, resonance frequency and quality factor on the tuning parameter. Notably, the described architectures provide a high range of tunability with frequency modulation above 30% combined with high Q-factors above 300, making them advantageous for telecom applications.

## 2. Materials and Methods

The proof-of-concept of reconfigurable devices resonating up to 12.55 GHz is based on numerical calculations that rely on simulation platform COMSOL Multiphysics^®^ 5.6 to analyze three different layouts of resonant circuits focused on double SRRs. The first two configurations show devices where the inner ring is subject to extrusion along the rings’ axis and mutual rotation, respectively; the third layout refers to the embedding of a double SRR onto a microelectromechanical system [58] to evaluate the electromagnetic response at microwaves after mechanical deformation of the piezoelectric structure. The modeling is performed with the RF module by considering total field formulation of electromagnetic waves in frequency domain, for which a sufficiently wide perfectly matched spherical layer is required to set a far boundary condition for the electromagnetic signal. Two coaxial cylinders on both sides were introduced in the CAD layout to mimic the final part of SMA connectors employed for transmission measurements while reducing the computational requirements; then, we defined two lumped ports for the determination of scattering parameters during 0 dBm excitation. The dielectric characteristics of the materials are directly extracted by COMSOL standard library. Angular or linear displacement was imposed by parametric sweeping during study of eigenfrequency for the first two configurations here investigated. This setting gives the possibility to adopt a complete physics-controlled mesh, which consists of ~10^5^ volume elements, ~10^4^ surface elements and ~10^2^ elements on the sides. The third examined layout was treated by using different physical interfaces such as “Solid Mechanics”, “Electrostatics” and “Deformed Geometry” for determination of piezoelectric effect and mechanical deformation, and “Electromagnetic Waves—frequency domain” as mentioned above. Each study in the frequency domain has been performed after the convergence of the mechanical simulation. For each geometry, the frequency has been swept around that of the fundamental mode. The starting geometry and the deformed one was fully user-defined meshed and consisted, on average, of 10^6^ volume elements, 2 × 10^5^ surface elements and 2 × 10^4^ elements on the sides.

## 3. Results

### 3.1. Tuning by Linear Displacement

**Figure 1:**
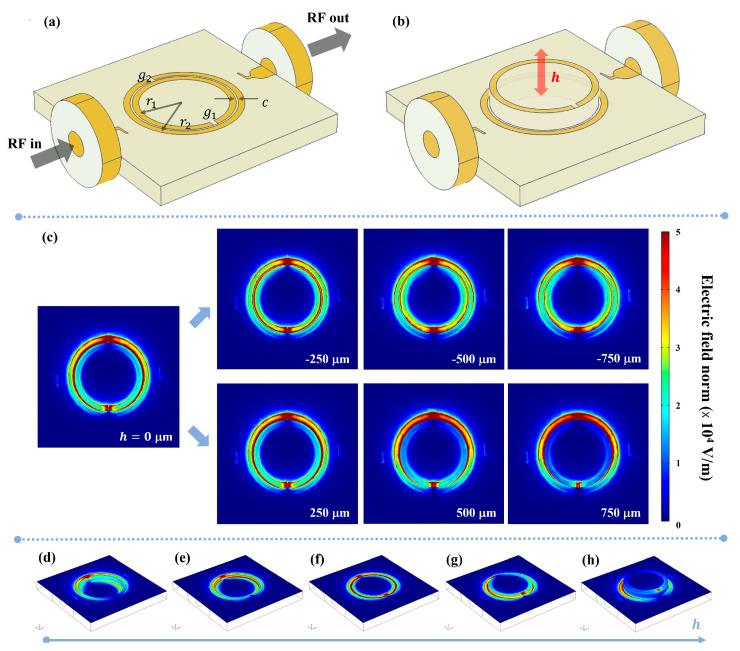
(**a**) Color rendering 3D CAD of the whole device drawn with COMSOL^©^ Multiphysics; the main features are indicated in black. (**b**) Sketch of the DSRR upon extrusion of the inner ring. (**c**) Left: Electric field map for coplanar rings (h=0 μm). Right: Top view of electric field distribution at resonance frequency as a function of extrusion h. (**d**) Isometric view of electric field distribution on rings planes for h=−750 μm, h=−250 μm (**e**), h=0 μm (**f**), h=250 μm (**g**), h=750 μm (**h**).

The first implementation includes MEMS-actuated linear displacement. Specifically, we investigated DSRRs with the structure reported in Figure 1a. The geometry is composed by two concentric Cu rings and two coplanar feedlines on top of a FR4 substrate of thickness $t_{s}$ = 1 mm. Two antennas are arranged to perform enhanced magnetic coupling and two coaxial ports are anti symmetrically placed with respect to the resonator center. The substrate dimension is 10 mm × 10 mm, while the two rings have mean radii of $r_{1}$ = 2.25 mm and $r_{2}$ = 2.65 mm, a width w of 300 µm, a coupling gap c of 100 µm and slits $g_{1}=g_{2}$ = 250 μm opposite to each other. Considering device sizes and resonance frequencies, a near-field radiation regime can be considered for the electric field [59].

With this first layout, we evaluated the electromagnetic response of a DSRR upon a parametric extrusion of the inner ring by variable height h, as schematically illustrated in Figure 1b. Specifically, the inner ring extrusion was carried out above and below with respect to the condition of rings coplanarity, with steps of 50 µm and total excursion of 1.5 mm, evaluating transfer functions and radiating near electric field at fundamental mode.

The selected two-dimensional maps of the electric field magnitude are reported in Figure 1c as top views. This means that the figure represents the electric field on a composite surface and the shown map displays colors according to electric field values at resonance frequency in the fixed or extruded ring planes depending on the location of the pixel on a specific ring. Specifically, for the areas associated with the outer ring and outside it, the field is taken on the (fixed) plane of the outer ring, while the field is taken on the (movable) plane of the inner ring and inside it.

For further detail, we report isometric views of electric field distribution on selected surfaces (Figure 1d–h). The RF electric field is confined in proximity of the slits and between the two rings when they are coplanar (red color portions). For extrusion h≠ 0, the field distribution in the inter-ring zone changes remarkably. The electric field is always higher near the two slits of each ring. When the rings are coplanar, there is another region of high, uniform field in between. Upon extrusion, the field between the two rings is no longer symmetric, and the confinement of the electric field for each single ring is accentuated; in fact, for the major extrusions here considered, each ring manifests an antinode near the respective slits and a diametrically opposite node. Differently from the configuration in which the inner ring is lowered, the maximum upward extrusion exhibits a reduction in the electrical field intensity in proximity of the inner ring slit up to 30%.

**Figure 2:**
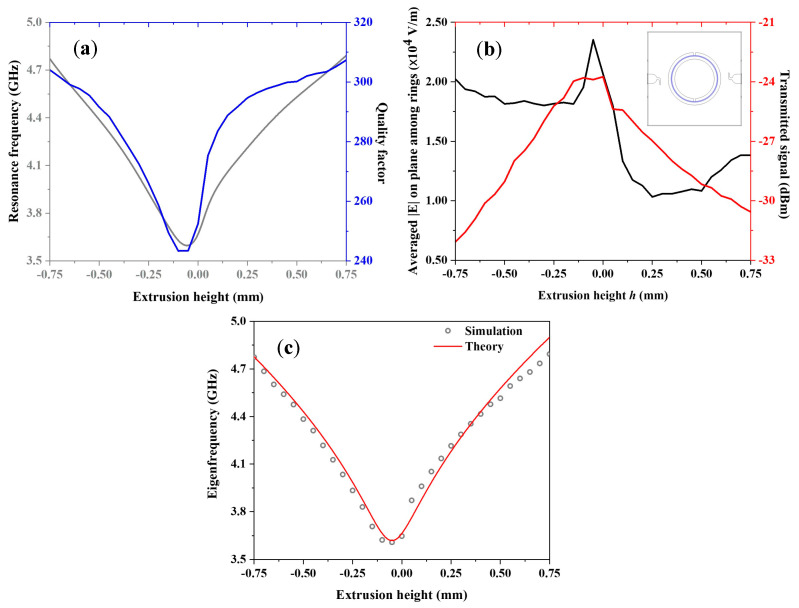
(**a**) Eigenfrequency (grey curve) and quality factor (blue one) of device as a function of amplitude of extrusion among the two rings. (**b**) Mean electric field norm on surface among the two rings (black line) and transmitted signal from device (red curve) depending on extrusion between rings. Inset: selected area for calculation of mean electric field norm. (**c**) Computed and analytic values of DSRR eigenfrequency depending on extrusion height.

In Figure 2a, we report the eigenfrequencies and the quality factor of DSRR as a function of the extrusion: both grow with (absolute) extrusion, though showing a slight asymmetry in upward and downward extrusion due to the different dielectric environments (i.e., air or FR4). Resonance frequency shows a minimum for h= −50 µm, namely 3.608 GHz, then increasing up to 4.790 GHz, corresponding to a total frequency modulation greater than 30% in the investigated range. The DSRR quality factor shows a variation of almost 20%. Figure 2b reports the transmitted signal (referring to scattering parameter $S_{21}$) and the averaged electric field norm calculated on the surface among the rings (as shown in the inset). The transmitted signal peaks for h= −50 µm, in agreement with transmitted signal profile.

For comparison, the DSRR eigenfrequency can be estimated for an azimuthal displacement of the inner ring by the following equation [60]:

(1) $$f_{0}=\frac{1}{2\pi \sqrt{LC}}=\frac{1}{2\pi \sqrt{2L_{T}\left[\frac{\left(\pi \left(r_{1}+w/2\right)-g\right)C_{pul}}{2}+\frac{\varepsilon_{0}wt_{s}}{2g}\right]}}$$

which relates the resonance frequency of conventional DSRR to the geometrical characteristics of the device. Here, $L_{T}$ indicates the total inductance of the device, proportional to $l\left(2.303\mathrm{Log}\frac{4l}{c^{\prime }}-\gamma \right)$, where l is the total mean length of the DSRR and γ is a geometry constant, equal to 2.451 for a circular SRR [61]; c′ is the coupling gap among rings. $C_{pul}=\frac{\sqrt{\varepsilon_{eff}}}{c_{0}Z_{0}}$ is the per unit length capacitance between rings, with effective permittivity of medium $\varepsilon_{eff}$, $c_{0}$ speed of light, $Z_{0}$ is the impedance of medium and $t_{s}$ is the substrate width. When the circuit width w is smaller than the substrate thickness $t_{s}$, $\varepsilon_{eff}=\frac{\varepsilon +1}{2}+\frac{\varepsilon -1}{2}\left[\frac{1}{\sqrt{1+12\left(t_{s}/w\right)}}+0.04{\left(1-w/t_{s}\right)}^{2}\right]$ and $Z_{0}=\frac{60}{\sqrt{\varepsilon_{eff}}}\ln\left(8\frac{t_{s}}{w}+0.25\frac{w}{t_{s}}\right)$ [62,63]. In our case, $\varepsilon_{eff}=3$ and $Z_{0}=115\;\Omega$. Finally, in our case we consider the coupling gap c′ dependent by extrusion height h: $c^{\prime }=\sqrt{c^{2}+h^{2}}$. Figure 2c compares the eigenfrequencies extracted by FEM simulation with those estimated by means of the analytical equation, showing good agreement.

This investigation demonstrates the efficiency of the linear displacement layout in SRR response modulation (up to 30% in the resonance frequency), and this effect is useful for frequency switching and signal filtering in telecommunications. Notably, during the mutual displacement of the rings, the quality factor of the whole device remains of the same order of magnitude despite the wide eigenfrequency variation, and the transmitted signal is attenuated by a maximum of 6 dB during the handling of the rings and a limited attenuation of 6 dB of transmitted signal when actuation is on. These two aspects represent two advantages for the use of the device in the telecommunication field because the noise is limited and the transmitted signal is not drastically reduced, respectively.

### 3.2. Tuning by Angular Displacement

**Figure 3:**
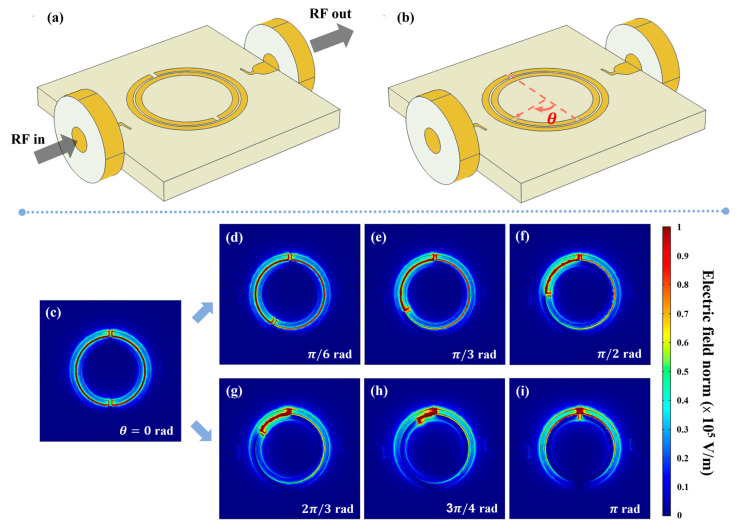
(**a**) Illustration of the whole device. (**b**) Representation of DSRR configuration with θ rotation of inner ring. (**c**,**i**) Map of electric field magnitude on the ring plane at resonance frequency vs. angle θ variation. Below: electric field distribution for θ=0 (**c**), π/6 (**d**), π/3 (**e**), π/2 (**f**), 2π/3 (**g**), 3π/4 (**h**) and π rad (**i**).

The second investigated layout includes a MEMS-actuated mutual rotation, which sets an angular displacement between the radial slits axis in the two rings, as illustrated in Figure 3a,b. Specifically, the inner ring has been rotated by a variable clockwise angle θ (by 5° steps) with respect to the outer. All the other parameters have been conserved with respect to the previous configuration. The magnitude of the near-field on the ring plane is shown for θ= 0° in Figure 3c and for other angular values in Figure 3d–i. The field intensity is maximum within the slits and in the inter-ring region joining the slits themselves, due to the coupling between the open ends of different slits. In the two limit cases, the electric field distribution is uniform in this region for θ= 0°, while for θ= 180° it is mainly confined near the slits position.

**Figure 4:**
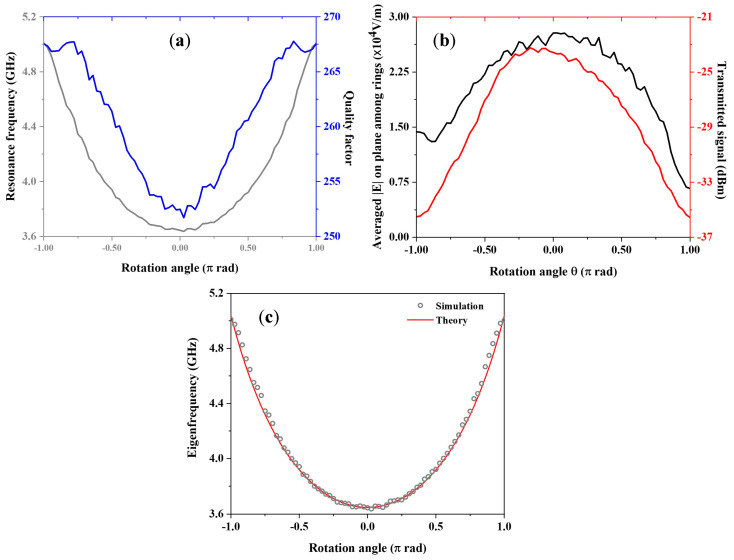
(**a**) Eigenfrequency (grey curve) and quality factor (blue one) of device vs. relative rotation between the two rings. (**b**) Mean electric field magnitude on the surface between the two rings (black line) and transmitted signal from device (red curve) as a function of rotation angle. (**c**) Numerical and analytical calculation of resonance frequency of angle tuned DSRR.

The DSRR resonance frequency and quality factor as a function of the rotation angle θ are shown in Figure 4a, while Figure 4b reports the averaged electric field magnitude on the ring plane and the transmitted signal. The eigenfrequencies vary rather symmetrically with respect to the rotation angle (and monotonically with respect to the absolute angular displacement) with 39% total modulation, while the maximum variation of the quality factor is 5%, much lower than the values reported for linear displacement modulation. The mean electric field magnitude in the coupling area is maximum near θ=0, as visible in the maps.

In this case, the dependence of resonance frequency $f_{0}$ from rotation angle θ can estimated by [64]:

(2) $$f_{0}=\frac{1}{2\pi \sqrt{2\left(r_{1}+w/2\right)L_{T}\frac{{\left(\pi +k\right)}^{2}-\theta^{2}}{2\left(\pi +k\right)}C_{pul}}},$$

where $k=\frac{C_{g}}{(r_{1}+w/2)C_{pul}}$, with slits capacitance $C_{g}=\frac{\varepsilon_{0}wt_{c}}{g}$, in which $t_{c}$ represent thickness of the copper layer. As for the rest, the slits $g_{1}$ and $g_{2}$ are aligned, and the capacitances per unit length in the two gap areas delimited by slits and rings are equal; as mutual rotation occurs, the two areas subtended by angle θ change, which leads to a decrease in the capacitance related to $g_{2}$ and an increase in that related to $g_{1}$. 

Comparison among our simulations and the analytical predictions are reported in Figure 4c and show a good agreement also in this case.

### 3.3. MEMS-Based Reconfigurable DSRR for Linear Extrusion

In the previous sections, the effectiveness of controlling the DSRR response by modifying its geometry has been demonstrated theoretically. The next step is to investigate the feasibility of tuning a DSRR embedded into manufacturable/available microelectromechanical actuators (or MEMS). In other terms, one should verify whether and to what extent can a MEMS deform a ring oscillator built upon it in a controlled fashion. A mechanically deformable SRR can be manufactured with standard MEMS fabrication techniques: MEMS generally include deformable elements—for instance, silicon and silicon compounds—which cause other elements, such as membranes, to move. In turn, thin layers of different materials, such as metals, can be deposited onto the membranes, to provide the structure with tunable electromagnetic behavior. Piezoelectric actuation is often employed (instead of electrostatic and electromagnetic alternatives) due to its high energetic efficiency. Applying small voltages to the piezo elements can produce comparably large structure deformations. Below, we show that, in MEMS-based ring oscillators, these in turn correspond to remarkable frequency modulation.

Let us examine MEMS’ capacity of ‘vertical’, i.e., normal to substrate, displacement, which can be manufactured on a silicon substrate out of a stack of materials, in turn made, for example, of a thin layer of polysilicon covered by another thin layer of tetraethyl orthosilicate (TEOS). If the substrate is hollow, with a cylindrical cavity running through its length, a part of the thin layer above becomes a suspended membrane, capable of (mainly vertical) deformation. The actuation can be obtained fabricating patterned thin sandwiches of materials above the membrane, each made of a lower and an upper electrode separated by a piezoelectric layer. Applying a voltage to the electrodes produces a deformation of the patterned structure and of the portion of membrane attached. Symmetric deformations of a circular membrane can be obtained patterning ring-shaped actuators, possibly built in separate portions separated from each other by small angular gaps, to address the stiffness of the piezo actuators. Additionally, better control is obtained if two (or more) concentric ring-shaped series of actuators are patterned. Other constructive details are given in ref. [58]. In the case of concentric rings, the membrane actuation will produce a vertical displacement, with consequent significant modification of the electromagnetic coupling.

**Figure 5:**
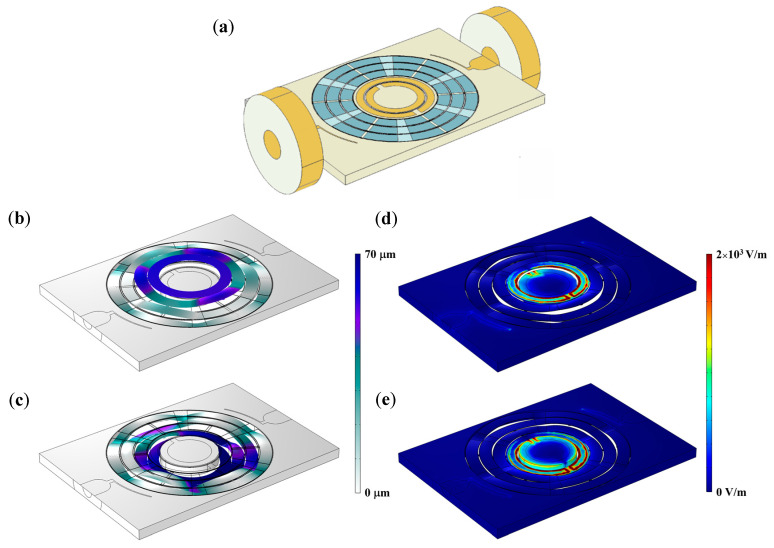
(**a**) False color 3D CAD of DSRR embedded in mechanical system (partially highlighted by blue shades), when no voltage is applied. Upward (**b**) and downward (**c**) solid displacement after application of 5 V voltage. (**d**) Electric field magnitude distribution at resonance frequency of 12.275 GHz after MEMS device actuation with 50 V for upward extrusion. (**e**) Electric field magnitude distribution on downward-displaced structure resonant at 12.525 GHz; DC voltage applied: 30 V.

The whole layout is schematically shown in Figure 5a. For this configuration, the DSRR has smaller radii than in the previous simulations, so that the whole device is contained in a 1 cm × 0.7 cm chip. Here, $r_{1}$ and $r_{2}$ are 1.05 mm and 1.45 mm, respectively, both gaps are 100 µm wide and arranged at the antipodes, while the width is 300 µm; antennas are longer than the previous configurations’ ones in order to increase the coupling with the connectors. As usual, the transfer function will depend on the geometry of the oscillator. In principle, the electrodes used for piezo actuation could be fed with AC signals (i.e., at RF) through capacitively coupled outer leads; however, we preferred to exploit coaxial connectors. The structure deformation results from the alternate actuation of different piezoelectric layers distributed along three concentric rings around the DSRR. The actuation in turn is controlled by the application of a DC voltage on dedicated piezo layers. Figure 5b,c shows the response of the MEMS to a 5 V actuation for upward and downward extrusion, respectively; the color bar refers to the absolute extrusion of each substructure. As can be seen, the height of the outer ring with respect to the FR4 substrate is almost constant. Then, on average, the ring can be approximated as lying on a plane parallel to the substrate itself.

**Figure 6:**
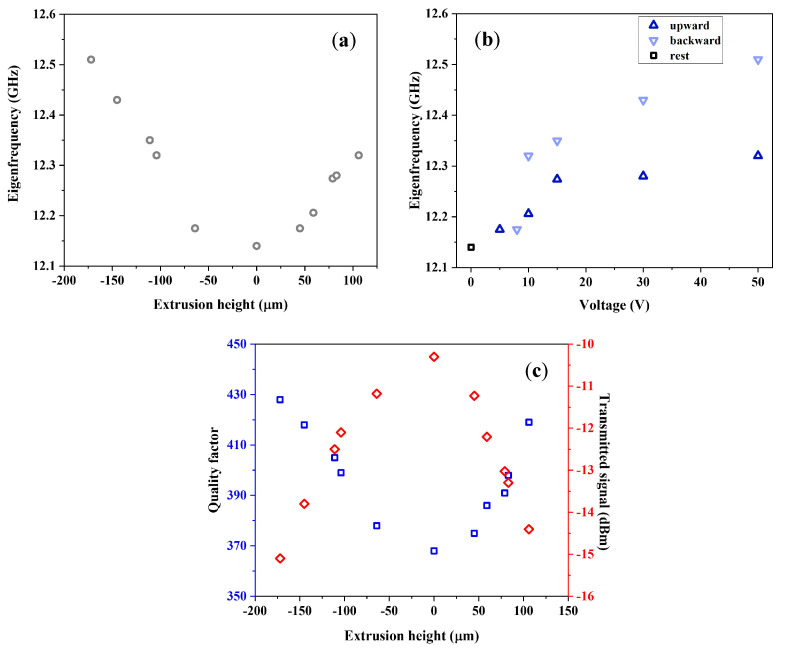
(**a**) Resonance frequency of the DSRR embedded in the MEMS chip as a function of extrusion height. (**b**) DSRR eigenfrequency vs. applied DC voltage. (**c**) Quality factor (blue squares) and insertion loss (red rhombuses) as a function of extrusion of MEMS-based ring oscillators.

Two cases of electric field distribution are reported in Figure 5d,e both with the DSRR in resonance. Figure 5d shows the electric field magnitude when the device is extruded upward upon application of 50 V DC voltage, at the eigenfrequency of 12.275 GHz, while Figure 5e shows the electric field magnitude with the outer ring extruded downward upon application of 30 V; the resonance frequency, here, is 12.525 GHz. In both cases, the field confinement at resonance is evident in regions next to DSRR, including slits. The eigenfrequency vs. extrusion are reported in Figure 6a; here, the eigenfrequency with MEMS at rest is 12.14 GHz, and the frequency shift obtained for the maximum computed extrusion of 170 μm is 370 MHz. The eigenfrequency vs. applied voltage is shown in Figure 6b, where up-pointing (down-pointing) triangles indicate DSRR eigenfrequencies depending on voltages applied for upward (downward) deformation. The black square point indicates the DSRR resonance with the MEMS at rest. The voltage is applied between the upper surface and lower piezoelectric surface—the latter used as a reference. From Figure 6a,b, it is clear that DSRR extrudes in a nonlinear way. Extrusion occurs more efficiently downward than upward. Downward extrusion is more accurately controlled, and the frequency shift range is broader.

In terms of practical operation, the initial voltage range (up to 15 V) could be employed since it accounts for most of the change. A further reduction in the control voltages can be achieved by optimizing the piezo materials beyond the standard parameters available in the COMSOL library. This can open the way to reach voltage values below 10 V, easily accessible on prototyping/PCB boards. Still in terms of operational performance, the most relevant parameter is the combination of a large range of tunability and a high Q factor since the last one sets the readout/filter band. The trend of quality factor and insertion loss of the whole device following extrusion of the outer ring are reported in Figure 6c, as blue squares and red rhombuses, respectively. Notably at rest, the MEMS-based reconfigurable DSRR is characterized by a quality factor around 400, a value like the cases of the device discussed in the previous sections. This Q-factor grows as the absolute value of the extrusion itself increases, until reaching a maximum variation of 16% for the examined range. Similarly, the transmitted signal changes as a function of the extrusion height, with a signal loss reduced by 6 dB on the full range.

Electromagnetically induced thermal effects on the device performance are expected to be negligible in the considered range of operating input powers. Finally, it is worth noting that for further accuracy in calculating the electromagnetic response of the system, it can be useful to consider the effect of potential alteration of the dielectric properties of constituent materials as a consequence of the induced deformation of the system [65]. Such effects can be accounted for in the model by modifying the dielectric function of the constituent materials, i.e., considering a non-zero coupling factor in the strain–charge formulation of the relative permittivity [66].

## 4. Discussion

Recently, there was a large interest and progress in the field of actively MEMS-based tunable circuits and metamaterials [1]. A first comparison with the literature and current state-of-the-art can be conducted in terms of frequency range of operation. Significant advances have been obtained for tunable metamaterials that work at THz frequencies up to the infrared [1]. Our study focused instead on SRR metamaterials resonating in the MW range, and specifically from the S band to the K band. This range is of significant interest for present and future telecommunication technologies.

The geometric and electromagnetic characteristics of the three analyzed layouts are summarized in Table 1. Notably, the dimensions of the examined resonators are compatible with standard CMOS manufacturing techniques. Thus, we expect a feasible integration in compact and robust monolithical devices operating as reconfigurable split ring resonators by MEMS-driven geometrical tuning.

To the best of our knowledge, no theoretical model describing the modulation of eigenfrequency as a function of extrusion of a ring along its axis had been presented before. Moreover, the two initial candidate deformable configurations provide a bandwidth of at least 1.1 GHz on a resonance frequency of 3.6 GHz, which corresponds to a frequency modulation over 30%. These performances are the state-of-the-art and even superior to those of comparable reconfigurable devices. In order to compare the performance of our architectures with the state-of-the-art, Table 2 displays features of SRRs reconfigurable by various types of actuators (such as varactor diodes, RF-MEMS, organic transistors, nematic liquids and magnetic switches) and whose first eigenfrequencies are included up to *K* band. In this frame, our devices exhibit the best loaded quality factor that reaches several hundreds, and a modulation percentage that exceeds 30%, at least for the two first layouts here analyzed. These aspects are relevant in view of the use of the device as a reconfigurable filter. In this respect, it is worth noting in terms of modulation performance that around 30% have been reported using liquid metals or pneumatic membrane actuators [67,68], but they are more complex to operate and monolithically integrate than MEMS-based technologies. In ref. [69], a similar performance was obtained combining E-coupled LC resonators with varactor diodes but the Q factor was below 100 in this case. Concerning the use of MEMS as actuators, the architecture described in ref. [70] employs cantilever MEMS and, in this respect, our configuration can be expected to be more robust. Furthermore, in both ref. [70] and [71], the Q factor is below 100 and in terms of operational performance the most relevant parameter is the combination of a large range of tunability and a high Q factor since the last one sets the read out band. Considering all these aspects, we conclude that the proposed design is advantageous for practical exploitation.

## 5. Conclusions

In the present work, we propose the tuning of MW signals by the reversible control of a conventional DSRR geometry. In this regard, we illustrated two candidate deformable configurations based on the linear displacement and mutual rotation of resonators, through which filtering travelling signals in a frequency span even on the order of hundreds of MHz was performed, and then presented the analytical models which predict resonant frequency distribution for both the configurations. Moreover, we suggest a compact reconfigurable resonant circuit based on the combination of a DSRR with a commercial MEMS layout, for the novel dynamic modulation of MWs. The embedding of resonator in piezo actuator chip developed for micro-motion in an azimuthal way show a shift of the resonance frequency of 370 MHz by the extrusion of only 170 μm, by the application of a static potential difference. This tunability range is combined with a high Q-factor (around 400) and a resonance bandwidth of 27 MHz. Thus, the whole range is able to accommodate 13 differently tuned operational frequencies. The MEMS technology for the proposed architecture is mature enough to guarantee reproducible results at operational voltages accessible on prototyping/PCB boards. By considering the compatibility with CMOS technologies, this makes the devices suitable for integration in low-power mobile communication devices and for the growing field of the Internet of Things (IoT), one of the most relevant for the industry of electronics, where the capability of easily tuning the frequency could be of help in variable environmental conditions. IoT devices operating in harsh environmental conditions could require energy scavenging, for which these devices could also prove useful—in principle, their tunability could help optimize the scavenging efficiency. Analogous remarks apply to wireless energy transfer applications. Finally, we emphasize that a similar concept and approach can also be applied in the optical regime, i.e., implementing miniaturized toroidal nanostructures such as rings, disks or nanoholes resonating in the visible range [79,80] and combined with piezoelectric actuators. In this respect, it is worth noting that the availability of optomechanical metamaterials with tunable responses/plasmons is of large interest from a classical to a quantum point of view, and can be expected to strongly impact applications including classical and quantum sensing and involving plasmons coupled to trapped molecules or entangled with photons [81].

## Figures and Tables

**Table 1:** Parameters of the investigated design geometries.

Displacement	Substrate Dimension (mm^2^)	$r_{1}\;$ (mm)	$r_{2}\;$ (mm)	c (µm)	$g_{1},g_{2}\;(µm)$	w(µm)	Rest Resonance Frequency (GHz)	Bandwidth (GHz)	Minimum Quality Factor
**Linear**	10 × 10	2.25	2.65	100	250	300	3.642 GHz	1.148	250
**Angular**	10 × 10	2.25	2.65	100	250	300	3.642 GHz	1.364	250
**Linear** **(with MEMS)**	10 × 7	1.05	1.45	100	100	300	12.140 GHz	0.370	380

**Table 2:** Features of reconfigurable MW ring resonators by dynamic tuning both excerpts from literature and shown in our study. The three dots in cells indicate that related values cannot be accurately extracted.

Ref.	Device	Actuator	Rest Resonance Frequency	Modulation	Bandwidth	Minimum Quality Factor
[69]	E-coupled LC resonators	Varactor diode	4.45 GHz	27% Continuous	1.19 GHz	<100
[72]	DSRR	Varactor diode	2.42 GHz	12% Continuous	280 MHz	<100
[73]	CTSSR	p-i-n diode switch	6.8 GHz	1.5% Discrete	100 MHz	10
[74]	SSRs	Organic electrochemical transistor	543 MHz	4% Continuous	22 MHz	…
[75]	CDSSR	BPF	7.8 GHz	Discrete	…	5
[70]	DSSR	Cantilever MEMS	10 GHz	15% Continuous	1.5 GHz	<100
[71]	TSSR	MEMS switches	6.685 GHz	2% Discrete	112 MHz	<100
[67]	DSRR	Pneumatic membrane	3.2 GHz	28% Continuous	890 MHz	<100
[68]	SSR	Liquid metal	3.52 GHz	30% Continuous	1.05 GHz	70
[76]	SRR	Nematic liquid crystal	6.5 GHz	12% Continuous	750 MHz	<100
[77]	JSRR	SQUID	11.5 GHz	11% Continuous	1.25 GHz	…
[78]	SSRs	Magnetic switch	…	Selective	…	…
**Our work**	DSRR	MEMS	3.642 GHz 3.642 GHz 12.4 GHz	**31%** **38%** 3 %	1.145 GHz 1.364 GHz 370 MHz	**250** **250** **380**

## Data Availability

Data are contained within the article.
